# Intensification with Intravenous Ustekinumab in Refractory Crohn’s Disease

**DOI:** 10.3390/jcm13030669

**Published:** 2024-01-24

**Authors:** Cristina Suárez Ferrer, José Arroyo Argüelles, Jose Luis Rueda García, Laura García Ramírez, Eduardo Martin Arranz, María Sánchez Azofra, Joaquín Poza Cordón, Jesús Noci Belda, Maria Dolores Martin-Arranz

**Affiliations:** 1 Inflammatory Bowel Disease Unit, Digestive Medicine Service, Hospital Universitario La Paz, 28046 Madrid, Spain; jmarroyo94@gmail.com (J.A.A.); eiiclapaz@gmail.com (L.G.R.); emarranz@salud.madrid.org (E.M.A.); maryazofra@gmail.com (M.S.A.); mmartinarranz@gmail.com (M.D.M.-A.); 2 IdiPAZ Study Group for Immune-Mediated Gastrointestinal Diseases, 28049 Madrid, Spain; 3 Faculty de Medicina, Universidad Autónoma de Madrid, 28049 Madrid, Spain

**Keywords:** Crohn’s disease, ustekinumab

## Abstract

Background: The rates of clinical and biochemical responses in Crohn’s disease (CD) patients treated with intravenous (IV) ustekinumab (UST) intensification are scarcely described. Methods: Patients with diagnosis of CD who were under intensified IV ustekinumab treatment (130 mg every 4 weeks) were retrospectively included, evaluating the clinical and biochemical response 12 weeks after the change in treatment regimen (switch from SC to IV), as well as the serum levels of the drug. Results: Twenty-seven patients, all of whom had transitioned to intensified intravenous ustekinumab treatment due to a secondary loss of response to the drug, were included in the retrospective analysis. At the baseline visit, prior to changing IV UST, differences in levels were observed between intensified and non-intensified patients (7216 vs. 2842 ng/mL, *p* = 0.00005). However, no significant differences were found between these two groups 12 weeks after IV intensification (7949 vs. 7937 ng/mL; *p* = 0.99). In patients with previous intensified UST SC, a decrease in fecal calprotectin was observed 12 weeks after starting IV intensification, going from a mean of 1463 ug/g to 751 ug/g, although the differences were not significant (*p* = 0.14). Conclusion: In our experience, intensifying treatment with IV UST leads to clinical and biochemical improvements in CD patients with a secondary loss of response to SC maintenance with this drug, and an increase in drug levels was observed 12 weeks after IV UST intensification.

## 1. Introduction

In recent years, we have witnessed a rapid development in the therapeutic arsenal for the management and treatment of inflammatory bowel disease (IBD).

The first anti-TNF drugs were introduced in 2002 and remain a fundamental pillar for the treatment of our patients, being the most commonly used biological drugs. More recently, other biologicals with different therapeutic targets have been approved for use, such as Vedolizumab (an anti-integrin drug) or ustekinumab (anti-IL-12 and IL-23) [1].

Undoubtedly, this variety of treatments has expanded our treatment options, achieving better control of the disease with higher response rates and achieving deep remission in most of our patients.

However, the options to optimize biological treatments when there is a loss of response to the medication, that is, the intensification guidelines, are not clearly established, especially when we refer to more recent drugs such as ustekinumab or vedolizumab [2].

Ustekinumab is recommended in Crohn’s disease for patients who have had an inadequate response, a had loss of response, or are intolerant (mainly due to the occurrence of adverse effects) to conventional treatment or anti-TNF, or where there are medical contraindications. It has shown the ability to induce and maintain remission in some patients, reducing the symptoms and intestinal inflammation associated with the disease.

In other words, ustekinumab is proposed as a second-line biological treatment after anti-TNF failure (or first-line treatment if there is a contraindication to the immunomodulatory or anti-TNF treatment) [3]. However, in real-life studies [4], most of the patients treated with ustekinumab have previously failed an anti-TNF or even two or three biologicals. Despite that, the clinical response to Ustekinumab stands at around 50% by week 52, with clinical remission rates reaching up to 39%.

This means that patients receiving ustekinumab are often refractory to treatment [4] and in a scenario where therapeutic alternatives are more limited. This probably translates into a greater need for treatment intensification.

In a recent review that included a network meta-analysis [5], drugs targeting IL-23 (ustekinumab and risankizumab) were identified as a potentially more effective strategy in patients with previous exposure to TNF antagonists.

The need to intensify ustekinumab for Crohn’s disease arises primarily due to the loss of response to the drug. Over time, some patients might experience reduced effectiveness or diminished response to the standard dosage of ustekinumab. In such cases, intensifying the treatment involves increasing the dose or frequency of ustekinumab administration to regain a better therapeutic response and effectively manage the symptoms of Crohn’s disease.

Evidence on the usefulness of treatment intensification comes from some real-life studies, in which benefits have been reported by shortening the regimen every 4 weeks and, even in some cases, every 3 weeks or even with an individualized regimen according to experience [6].

In previous studies in real life [7], it has been observed that after intensifying the subcutaneous ustekinumab regimen to 90 mg every 4 weeks, remission rates of up to 31% and clinical response rates of 61% are achieved.

Likewise, previous studies have explored the effectiveness of reinduction with intravenous ustekinumab (to subsequently continue with the subcutaneous regimen). A significant decrease in the Harvey Bradshaw index has been identified (reduction by 2.4 points (*p* = 0.0034)) [8]. Similarly, with the intensified regimen performed subcutaneously every 4 weeks, not only an improvement in the clinical indices has been observed, but also a biochemical response, especially in those patients with greater underlying inflammatory activity.

However, not all patients achieve a response after shortening the administration interval [9], so it is necessary in the management of our patients to explore other alternatives for drug intensification, such as intravenous drug transfer.

The proactive determination of biological drug levels in IBD, although yielding controversial results in previous studies, seems to be associated with better disease control and improved long-term outcomes. However, this correlation between drug levels and efficacy has been established with antiTNF drugs, not with other biological medications.

However, monitoring ustekinumab levels in the blood in the treatment of CD [10] can help to determine the appropriate amount of medication needed to control disease symptoms and maintain long-term remission. Therefore, the interpretation of these levels may vary depending on the patients, and, currently, the therapeutic range has not been established.

The objective of our study is to analyze the clinical response, biochemical response, and endoscopic/ultrasound improvements after intensifying treatment with an intravenous regimen every 4 weeks. Likewise, the goal is to determine the change in drug levels in the blood after switching from ustekinumab to intravenous maintenance treatment and ultimately establish if there is a relationship between drug levels and drug efficacy.

## 2. Material and Methods

A study was conducted where patients with a previously established diagnosis of Crohn’s disease, undergoing treatment with intensified intravenous ustekinumab every 4 weeks, were retrospectively included. At our center, the cost of intravenous ustekinumab is lower than the subcutaneous form. Therefore, for those patients undergoing drug intensification every 4 weeks in clinical practice, there was a switch to the intravenous form of the medication.

Only those with stable follow-up in the IBD unit of Hospital La Paz were chosen, so it was possible to retrospectively assess the evolution and response to treatment.

Data related to patients’ baseline characteristics were collected. Likewise, data related to the patients’ previous pharmacological and surgical treatment for their IBD, as well as the previous ustekinumab regimen (start date, dose, and regimen), were included.

Clinical activity was assessed before switching to intravenous treatment, 12 weeks after the switch, and at the end of follow-up. The follow-up time was calculated from the date of the switch to intravenous treatment with ustekinumab every 4 weeks until the date of the last appointment. Clinical response was considered as a decrease in the Harvey Bradshaw score (HBI) [11] of ≥3.

Biochemical response was also assessed, as well as fecal calprotectin and C-reactive protein in blood, prior to the change to intravenous ustekinumab (considering the baseline level) and 12 weeks after the change to intravenous.

Finally, in those patients in whom it was available, endoscopic and radiological activities were investigated using intestinal ultrasound prior to the change to intravenous (baseline) and 12 weeks after it. Given the retrospective nature of the study, with the possible difficulty of having a baseline endoscopic and/or radiological study, examinations performed in the 6 months prior to the intravenous intensification of ustekinumab were included, provided that there were no clinical or treatment changes in this period. To assess the endoscopic response, the SES-CD index was used [11], considering remission ≤3 points.

As per usual clinical practice, in our center, trough levels of the drug (ustekinumab) are requested prior to each administration. Levels were assessed prior to switching to intravenous treatment (considering this as baseline levels) as well as after 12 weeks of the intensified regimen.

A descriptive analysis of the baseline characteristics and those related to their IBD was performed. For continuous variables, the mean and standard deviation were calculated; for the categorical, the percentages and 95% confidence intervals were calculated. Provided that the variables have a normal distribution (verified using the Shapiro–Wilk test), the categorical variables were compared using the c2 test, and the quantitative variables using the Student’s T test. Otherwise, the corresponding non-parametric test was applied. A value of *p* < 0.05 was considered statistically significant. The analysis was carried out using Stata version 16 for Mac.

## 3. Results

A total of 27 patients with Crohn’s disease receiving intensified treatment with ustekinumab, 130 mg intravenously every 4 weeks, were retrospectively included. All of them had undergone previous treatment with UST SC. Among them, five patients (18.5%) were receiving concomitant treatment with azathioprine.

Baseline and IBD-related characteristics of the included patients are summarized in Table 1.

Regarding previous treatment received by the patients, 4/27 (14.8%) were under treatment with UST on the first line, 10/27 (37%) on the second line, and 9/27 (33.4%) on the third line; 4/27 (14.8%) had failed three biologicals. The median number of previous biologicals was 2, IQR: 1–2.

The reason for intensification with IV UST was the secondary loss of response in all included patients, seven of whom (25.93%) were found in the context of post-surgical recurrence.

It should be noted regarding the subcutaneous regimen prior to switching to intravenous that 10 patients (37.03%) were already under treatment with intensified SC UST (9 patients every 4 weeks and 1 patient every 6 weeks), while the remaining 17 patients (62.96%) were under treatment with a standard schedule of SC UST every 8 weeks.

No adverse effects related to the use of intravenous ustekinumab were observed during the study.

**Figure 1:**
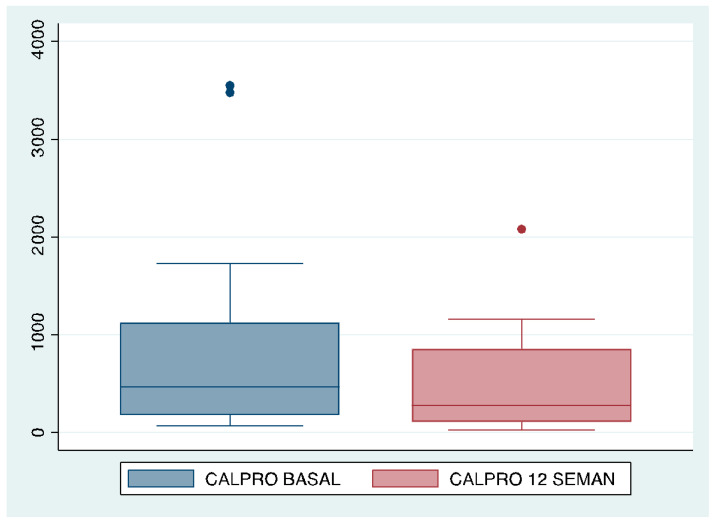
Improvement in fecal calprotectin levels 12 weeks after intravenous drug intensification.

When assessing the clinical activity of the included patients, 70% (19 patients) had baseline activity (HBI ≥ 5), with no clinically or statistically significant changes at 12 weeks of treatment and at the last follow-up visit (70%, 67%, respectively). However, if we consider the severity of the clinical activity, it is noteworthy that 10 patients (37%) had severe baseline activity (HBI ≥ 9), decreasing to 14.8% (4 patients) 12 weeks after switching to intravenous and 11% (3 patients) at the end of follow-up Figure 1.

Among the 17 patients without prior intensified treatment before the switch to intravenous, baseline activity was mild in 26.7%, moderate in 26.7%, and severe in 6.6%. In this group, at 12 weeks after the intravenous intensification, mild activity was observed in 60%, 13.3% had moderate activity, and no patient exhibited severe activity. However, these differences did not reach statistical significance.

**Figure 2:**
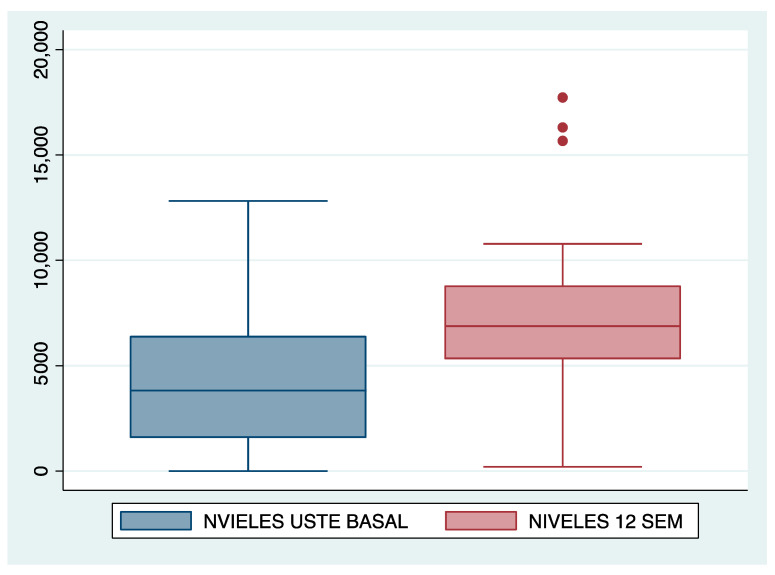
Improvement in ustekinumab levels 12 weeks after intravenous drug intensification.

Overall, taking into account all the patients, a reduction in fecal calprotectin levels was observed at 12 weeks (463 vs. 272.5 ug/g), *p* = 0.08 Figure 2.

In patients with intensified sc UST, fecal calprotectin decreased 12 weeks after starting IV intensification, from a mean of 1463 to 751 ug/g, although the differences were not statistically significant (*p* = 0.14).

Likewise, a reduction in CRP was detected after 12 weeks of IV treatment (6.6 vs. 4.1 mg/L), although statistical significance was not reached (*p* = 0.3).

**Figure 3:**
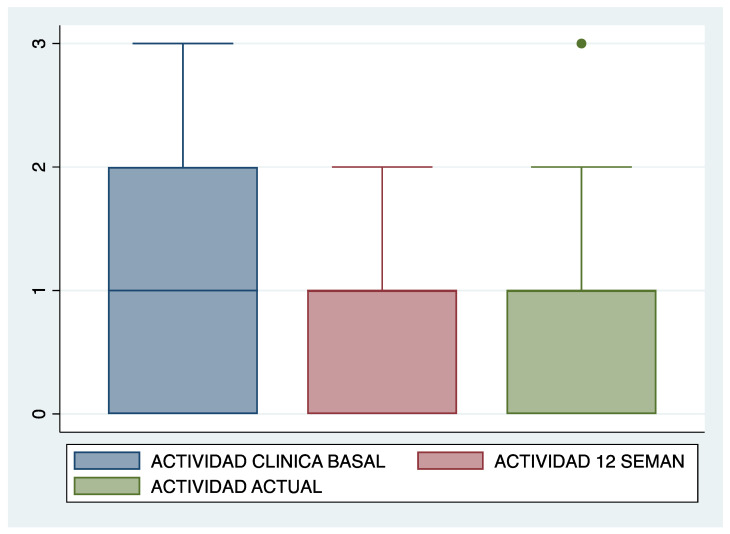
Clinical activity at baseline, 12 weeks after switching to ustekinumab iv, and at the end of follow-up.

A significant increase in ustekinumab levels was observed 12 weeks after intensifying intravenous treatment and shortening the regimen (in those patients who initiated treatment with ustekinumab every 8 weeks sc), 7216 vs. 2842 ng/mL (*p* = 0.0005) Figure 3.

The previous results are summarized in Table 2.

Despite these differences in drug levels after 12 weeks of IV treatment, no differences were observed among those patients who started with the intensified drug at the baseline visit (7949 vs. 7937 ng/mL; *p* = 0.99).

No correlation was found between higher drug levels at week 12 and the absence of clinical activity when analyzing the subgroup of patients without prior intensified drug regimens (8413.56 vs. 6672.5; *p* = 0.5).

Finally, the endoscopic study at baseline and at week 12 was available in only three patients. Two of them were in a situation of post-surgical recurrence and one with luminal activity. Endoscopic improvement was identified in two of the three patients, with a reduction in endoscopic activity indices (i3 at baseline vs. i1 at week 12, and SES-CD 26 vs. SES-CD 7).

As for ultrasound activity, it was available in 10 patients (at baseline and at 12 weeks), with significant improvements (no significant ultrasound activity) in 3 of them.

## 4. Discussion

Ustekinumab has been proven to be a safe and effective drug in patients with Crohn’s disease both in clinical trials [12] and in real-life cohorts [13,14,15,16], also demonstrating excellent treatment survival in patients responding to the drug [17].

However, there is little evidence on what to do if there is an inadequate response or the disease relapses during maintenance. A recent meta-analysis [18] showed that reinduction with UST or interval shortening may be effective therapeutic alternatives in this setting, especially in patients coming from a standard maintenance regimen of sc ustekinumab every 8 weeks.

It should be noted that our study not only investigated the possibility of intensifying the maintenance treatment period (every 4 weeks), but also of administering the medication intravenously. In addition, 37% of the included patients had already started an intensified ustekinumab regimen before switching to intravenous (subcutaneous every 4 or 6 weeks).

In our experience, we observed a clinical and biochemical improvement (reduction in fecal calprotectin and CRP levels) 12 weeks after switching to the intravenous regimen, even in those patients who switched from the intensified subcutaneous regimen. It should be noted that most of the studies on the subject assessed the effectiveness of treatment intensification solely with clinical criteria and not biochemical criteria as in our study [19].

We have previous evidence [20,21,22] on the possible relationship between blood drug levels and therapeutic efficacy. However, in the case of ustekinumab, this is an underexplored field. In our study, an overall increase in ustekinumab blood levels was observed after intravenous intensification, although these differences did not reach statistical significance in those patients who switched from the intensified subcutaneous regimen.

Ustekinumab has shown, in previous studies, not only clinical, but also endoscopic [23,24], ultrasonographic [25,26], and even histological [27] improvements. Despite this, studies addressing the efficacy of intensification strategies in the face of loss of response during maintenance often measure targets only with clinical or biochemical response. In the present study, we do not have power to draw conclusions in this regard. However, it should be noted that in a cohort of highly refractory patients, endoscopic improvement was quite common.

One of the main limitations of our study is the limited sample size. In fact, we cannot rule out, given the good results obtained, the absence of statistical significance being related to the sample size (type II error). More studies on a wider population are needed.

However, since this is a study in real clinical practice, increasing the sample size is complex, and the inclusion rate is unpredictable.

On the other hand, one of the main strengths to highlight is that, to our knowledge, this is the first study that addresses the possibility of intensifying the treatment, not only by reducing the regimen, but also by switching to intravenous administration as maintenance treatment, also obtaining good results and broadening the therapeutic possibilities in these patients.

## 5. Conclusions

In our experience, intensifying ustekinumab treatment, not only by reducing the interval (to 4 weeks) but also by transitioning to intravenous administration, is a safe and effective option for a significant proportion of patients.

When assessing the clinical activity of these patients, around 70% exhibited baseline activity, with no significant changes observed after 12 weeks of treatment. However, among those with severe baseline activity, a notable reduction was seen to 14.8% after 12 weeks of intravenous treatment. Although overall fecal calprotectin levels reduced at 12 weeks, statistical significance was not reached. Similarly, C-reactive protein (CRP) levels showed a decrease after 12 weeks of intravenous treatment, although this is not statistically significant.

Moreover, there was a substantial increase in ustekinumab levels after 12 weeks of intensified intravenous treatment in patients who initially received the drug every 8 weeks subcutaneously. Nevertheless, no differences in drug levels were observed among those who started with intensified drug treatment from the beginning. Endoscopic improvement was detected in a small subset of patients, while ultrasound activity showed significant improvement in three out of ten patients evaluated at both the baseline and 12 weeks.

Notably, no adverse effects were noted with the intravenous administration of ustekinumab during the study.

## Figures and Tables

**Table 1:** Patient baseline characteristics.

Variables	Number of Patients, %					
**Sex**	**Women**			**Men**		
	(12) 44.4%			(15) 55.5%		
**Tobacco**	**No**		**Yes**		**Former smoker**	
	(16) 59.3%		(6) 22.2%		(5) 18.5%	
**Extraintestinal manifestations**	**No**			**Yes**		
	(22) 81.5%			(5) 18.5%		
**Location (Montreal)**	**L1**	**L2**		**L3**		**L4**
	(18) 66.6%	(0), 0%		(6), 22.2%		(3), 11.1%
**Phenotype (Montreal)**	**B1**		**B2**		**B3**	
	(6) 22.2%		(15) 55.5%		(6) 22.2%	
**Perianal involvement**	**No**			**Yes**		
	(18) 66.7%			(9) 33.3%		
**Previous surgeries**	**No**	**1**		**2**		**3**
	(18), 66.6%	(4), 14.8%		(4), 14.8%		(1), 3.7%
**Concomitant immunosuppression**	**No**			**Yes**		
	(22), 81.4%			(5), 18.5%		

**Table 2:** Analysis of clinical activity, CRP, fecal calprotectin, and UST levels before and after (at 12 weeks) starting UST IV.

Variables	Basal				12 Weeks			
**Clinical activity (n, %)**	**No**	**Mild**	**Moderate**	**Serious**	**No**	**Mild**	**Moderate**	**Serious**
	8 29.6%	9 33.3%	8 29.6%	2 7.4%	9 33.3%	13 48.1%	5 18.5%	0 0%
**PCR**	**M_edian_**		**I.Q.R.**		**M_edian_**		**I.Q.R.**	
	6.6		10.7		4.1		6.7	
**Calprotectin**	**M_edian_**		**I.Q.R.**		**M_edian_**		**I.Q.R.**	
	463		946.5		272.5		749.5	
**UST levels**	**M_edian_**		**I.Q.R.**		**M_edian_**		**I.Q.R.**	
	3810		4840		6870		3510	

## Data Availability

The data is available in a locally anonymized file in case further studies are required in the future, and it will be preserved for a minimum of 4 years.
